# A new combination of 5’- and 3’-untranslated regions increases the expression of mRNAs in vitro and in vivo

**DOI:** 10.18699/vjgb-25-81

**Published:** 2025-10

**Authors:** D.N. Antropov, O.V. Markov, A.S. Dome, P.A. Puchkov, E.V. Shmendel, D.V. Gladkikh, V.M. Golyshev, A.M. Matveeva, M.A. Maslov, G.A. Stepanov

**Affiliations:** Institute of Chemical Biology and Fundamental Medicine of the Siberian Branch of the Russian Academy of Sciences, Novosibirsk, Russia; Institute of Chemical Biology and Fundamental Medicine of the Siberian Branch of the Russian Academy of Sciences, Novosibirsk, Russia; Institute of Chemical Biology and Fundamental Medicine of the Siberian Branch of the Russian Academy of Sciences, Novosibirsk, Russia; Lomonosov Institute of Fine Chemical Technologies, MIREA – Russian Technological University, Moscow, Russia; Lomonosov Institute of Fine Chemical Technologies, MIREA – Russian Technological University, Moscow, Russia; Institute of Chemical Biology and Fundamental Medicine of the Siberian Branch of the Russian Academy of Sciences, Novosibirsk, Russia; Institute of Chemical Biology and Fundamental Medicine of the Siberian Branch of the Russian Academy of Sciences, Novosibirsk, Russia; Institute of Chemical Biology and Fundamental Medicine of the Siberian Branch of the Russian Academy of Sciences, Novosibirsk, RussiaInstitute of Chemical Biology and Fundamental Medicine of the Siberian Branch of the Russian Academy of Sciences, Novosibirsk, Russia; Lomonosov Institute of Fine Chemical Technologies, MIREA – Russian Technological University, Moscow, Russia; Institute of Chemical Biology and Fundamental Medicine of the Siberian Branch of the Russian Academy of Sciences, Novosibirsk, Russia

**Keywords:** synthetic mRNA, RNA delivery, nucleotide modifications, untranslated region, lipid nanoparticle, синтетическая мРНК, доставка РНК, модификации нуклеотидов, нетранслируемая область мРНК, липидные наночастицы

## Abstract

mRNA vaccine technologies have been actively developing since the beginning of the 21st century and have received a major boost from new findings about the functioning of the immune system and the development of efficient vehicles for nucleic acid delivery. The mRNA vaccine demonstrates superior properties compared to the DNA vaccine, primarily due to accelerated mRNA vaccine development, enhanced flexibility, and the absence of integration into the genome. Artificial mRNAs have biotechnological and medical applications, including the development of antiviral and anticancer mRNA therapeutics. The effective expression of therapeutic mRNA depends upon the appropriate selection of structural elements. Along with the addition of the 5’-cap, appropriate polyadenylation, and sequence codon optimization, 5’- and 3’-untranslated regions (UTRs) play an important role in the translation efficiency of therapeutic mRNAs. In this study, new plasmids containing a novel combination of UTR pairs, namely 5’-UTR-4 and 3’-UTR AES-mtRNR1, were constructed to obtain artificial mRNAs encoding green fluorescent protein (GFP) and firefly luciferase (FLuc) with new structural elements and properties. The novel combination of the UTRs, which is described in this article for the first time, in addition to sufficient polyadenylation and pseudouridinilation of mRNA, was demonstrated to strongly increase the translation of codon-optimized sequences of reporter mRNAs. We generated lipoplexes containing the aforementioned reporter mRNAs and liposomes composed of cationic lipid 2X3 (1,26-bis(cholest-5-en-3beta-yloxycarbonylamino)-7,11,16,20-tetraazahexacosane tetrahydrochloride) and helper lipid DOPE (1,2-dioleoyl-sn-glycero-3-phosphoethanolamine). For in vivo experiments, the liposomes were decorated with 2 % of 1,2-distearoyl-sn-glycero-3-phosphoethanolamine-N-[amino(polyethylene glycol)-2000] (DSPE- PEG2000). The translation efficiency of mRNAs was found to be superior for the novel UTR combination compared with HBB gene UTRs, both in vitro and in vivo. When mRNA is administered intramuscularly, the proposed combination of UTRs provides lasting expression for more than 4 days. The results demonstrated that the novel UTR pair combination could be useful in the development of artificial mRNAs with enhanced translation efficiency, potentially reducing the dose for mRNA-based therapeutics.

## Abstract (ru)

Технология мРНК-вакцин начала активно развиваться в начале XXI в. и получила хороший стимул за счет расширения знаний о функционировании иммунной системы человека и успехов в синтезе вариантов молекул-доставщиков. Иммунизация с помощью мРНК-вакцин является более эффективной, чем иммунизация с помощью ДНК, благодаря более быстрой разработке, гибкости технологии и отсутствию интеграции в геном. В наши дни искусственные мРНК используют в различных биотехнологических и медицинских целях, включая разработку противовирусных и противораковых мРНК-вакцин. Для их эффективной экспрессии необходимо правильно подобрать структурные элементы мРНК. Помимо добавления в структуру мРНК 5’-кэпа, достаточного уровня полиаденилирования и оптимизации последовательности кодонов, 5’- и 3’-нетранслируемые области (НТО) играют важную роль в трансляционной эффективности терапевтических мРНК. В настоящем исследовании для получения искусственных мРНК были сконструированы плазмидные конструкции, содержащие в своем составе новую комбинацию нетранслируемых областей – 5’-UTR-4 и 3’-UTR AES-mtRNR1. Для новой комбинации НТО, впервые описанной в данной работе, было показано значительное увеличение уровня трансляции кодон-оптимизированных последовательностей репортерных мРНК, кодирующих GFP (зеленый флюоресцентный белок) и FLuc (люцифераза светлячка), содержащих в своем составе псевдоуридин и поли(А)-последовательность. В ходе работы были сформированы комплексы вышеупомянутых репортерных мРНК с липосомами, состоящими из катионного липида 2Х3 (1,26-бис(холест5-ен-3β-илоксикарбониламино)-7,11,16,20-тетраазогексакозан тетрагидрохлорид) и липида-хелпера DOPE (1,2-диолеил-sn-глицеро-3-фосфоэтаноламин). Для экспериментов in vivo в состав липосомальной композиции добавляли 2 % 1,2-дистеароил-sn-глицеро-3-фосфоэтаноламин-N-[амино(полиэтиленгликоль)-2000] (DSPE-PEG2000). Новая комбинация НТО продемонстрировала более высокую эффективность трансляции мРНК в сравнении с β-глобиновыми НТО как in vitro, так и in vivo. При внутримышечном введении мРНК предложенная комбинация НТО обеспечивает длительную экспрессию более четырех суток. Результаты исследования показали высокую эффективность новой комбинации НТО для повышения уровня трансляции искусственных мРНК, что может быть использовано для снижения терапевтической дозы мРНК в составе вакцин.

## Introduction

**Fig. 1.**
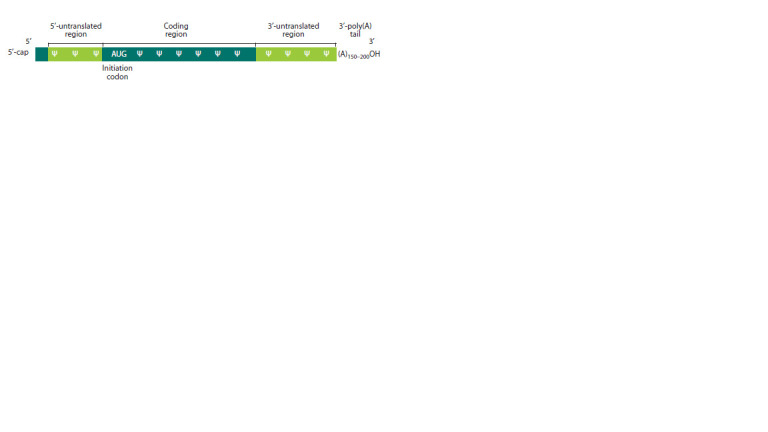
The mRNA design optimal for effective translation. Inserted pseudouridines are indicated by the “Ψ” sign.

mRNA vaccine technologies are emerging every year to defend humans from viral pathogens and even cancers. The COVID-19 pandemic proved the necessity of the fast development of vaccines targeted against a certain species of viruses. The release of different mRNA vaccines, such as BNT162b2, mRNA-1273, and others, has enabled effective vaccination of the population. It is clear that for effective and prolonged expression of antibodies, specific structure of mRNA is crucial (Fig. 1).

The necessity of UTRs in mRNA for various mRNA models is widely recognized (Chatterjee, Pal, 2009; Morais et al., 2021; Chen et al., 2022; Kirshina et al., 2023), along with such components as the Kozak sequence, which is required for translation (Kozak, 1989). The incorporation of 5′-UTR sequences enhances translation not only by protecting the coding sequences from nucleases but also by recruiting ribosomal machinery to a translation start site (Chatterjee, Pal, 2009). The most common method for enhancing translation is the addition of IRES (internal ribosome entry site) elements with complex secondary structure to the 5′ end of mRNA, which promotes the recruitment of translational complexes at various stages of translation (Andreev et al., 2009). Sample et al. (2019) have successfully identified a high-ribosome-load sequence of a 5′-UTR (designated as “5′-UTR-4”) using machine learning methods. The application of computationally selected sequences enabled the researchers to enhance translation intensity more than 100-fold, thus proving the efficacy of machine learning techniques.

3′-UTRs provide the mRNA molecule with a defense against nucleases, including the prevention of poly(A) tail degradation, which can influence the half-life of mRNA and the yield of the encoded protein. Derived from the human HBB gene, β-globin UTRs have been commonly used and demonstrated remarkable efficacy in both reporter and viral mRNA applications (Zhuang et al., 2020). The incorporation of a chimeric 3′-UTR called AES-mtRNR1, which comprises a part of 16S ribosomal rRNA of archaea (AES: amino-terminal enhancer of split) and a part of a mitochondrial rRNA (mtRNR1), allowed for 3-fold higher translation intensity in vitro in comparison to the standard β-globin 3′-UTR (Orlandini von Niessen et al., 2019). Their result was confirmed by in vivo application to a luciferase model (Orlandini von Niessen et al., 2019), indicating the universality of such a tandem UTR sequence. Thus, the previous investigations directed at the selection of the most effective UTR structures allow to design combinations and test their translational effectiveness

In this study, we introduced a novel combination of 5′- UTR-4 and 3′-UTR AES-mtRNR1 (hereafter, “synthetic UTRs”) into a single mRNA for the first time. We constructed reporter mRNAs, encoding GFP or FLuc, containing the novel combination of UTRs for delivery into human cells in vitro and live animal tissues in vivo. Cationic liposomes composed of the cationic lipid 2X3, the helper lipid DOPE, and DSPE-PEG2000 (for in vivo experiments) were used to deliver mRNAs. The expression of the reporter mRNAs with synthetic UTRs was demonstrated to be 5–6-times higher than that of β-globin UTRs in vitro and in vivo.

## Materials and methods

Plasmid design, construction, and synthesis. Sequence of the hyperactive T7 promoter was taken from (Conrad et al., 2020). Sequences of β-globin 5′- and 3′-UTRs and those of UTRs 5′-UTR-4 and AES-mtRNR1 (3′-UTR) (see Supplementary Table S1)1 were obtained from NCBI and (Andreev et al., 2009; Leppek et al., 2022), respectively. A multiple cloning site (MCS) between 5′- and 3′-UTRs was designed using several most popular unique restriction sites. Downstream of 3′-UTRs, an XbaI restriction site was inserted to generate a linearized DNA template for in vitro transcription.


Supplementary Materials are available in the online version of the paper: https://vavilovj-icg.ru/download/pict-2025-29/appx24.pdf


Fragments containing MCS and UTRs were synthesized by the Laboratory of Synthetic Biology at the Institute of Chemical Biology and Fundamental Medicine (ICBFM) SB RAS and cloned into the pCMV6-Entry vector (OriGene, USA; see Supplementary Fig. S1) by means of restriction sites Psp124BI and XmaI (SibEnzyme, Russia). Two parallel cloning reactions resulted in plasmid vectors: pCMV6_T7_bglob_AGG (containing 5′- and 3′-UTRs of the human HBB gene; see Supplementary Fig. S2) and pCMV6_T7_synth_AGG (containing 5′-UTR-4 and AES-mtRNR1; see Supplementary Fig. S3).

ORFs of GFP and FLuc were PCR-amplified from plasmids phMGFP (Promega, USA) and pCDH-EF1a-Luc2-IRESmKate2 (Yuzhakova et al., 2022), respectively, with primers containing restriction sites, the Kozak sequence, and start and stop codons and were cloned by the restriction–ligation method into plasmids pCMV6_T7_bglobUTR_AGG and pCMV6_T7_synthUTR_AGG.In vitro transcription and mRNA purification. GFP and FLuc with β-globin UTRs and the synthetic UTRs were obtained by in vitro transcription using T7 polymerase (Biolabmix, Russia). The Anti-Reverse Cap Analog (ARCA) (Biolabmix, Russia) and pseudouridine triphosphate (Biolabmix, Russia) were added during the transcription to modify mRNA structure. After the RNA synthesis, the DNA template was removed with DNase I (Thermo Fisher Scientific, USA). Poly(A) polymerase (New England Biolabs, USA) was used to polyadenylate 3′ termini of the synthesized mRNA by the standard protocol. The RNA products were purified via precipitation with 2.5 M LiCl followed by storage of the precipitate at −20 °C for 30 min and subsequent centrifugation at 16,000 × g for 15 min at 4 °C. The pellet was washed with 70 % ethanol and dried for 10 min at room temperature with subsequent dilution in diethylpyrocarbonate (DEPC)-treated H2O.Preparation of cationic liposomes. А solution of 1,26- bis(cholest-5-en-3β-yloxycarbonylamino)-7,11,16,20-tetraazahexacosane tetrahydrochloride (2X3; see Supplementary Fig. S4) in a CHCl3–CH3OH mixture (1:1, v/v) was added to a solution of 1,2-dioleoyl-sn-glycero-3-phosphoethanolamine (DOPE) in CHCl3 at a molar ratio of 1:3 and gently stirred. To obtain PEG-containing cationic liposomes, a solution of DSPE-PEG2000 (Lipoid, Germany) (2 % mol.) in CHCl3 was added to the 2X3-DOPE solution at a molar ratio of 1:3. Organic solvents were removed in vacuo, and the obtained lipid film was dried for 4 h at 0.1 Torr to remove residual organic solvents. Then, it was hydrated using deionized water at 4 °C overnight. The liposomal dispersion was sonicated for 15 min at 70–75 °C in a bath-type sonicator (Bandelin Sonorex Digitec DT 52H, Berlin, Germany), filtered (0.45 μm Chromafil® CA-45/25; Macherey–Nagel, Düren, Germany), flushed with argon, and stored at 4 °C.

Size and zeta-potential measurement. Lipoplexes were pre-formed via mixing of equal (25 μL) volumes of the RNA and liposome solutions at appropriate concentrations in saline (154 mM sodium chloride). Lipoplex formation was carried out for 20 min at 25 °C. Next, 10-μL aliquots of lipoplexes were diluted in 1 mL of DEPC-treated water. To measure physicochemical parameters, 1 mL of a lipoplex or liposome suspension was placed into a DTS1070 folded capillary cuvette (Malvern Instruments, Malvern, UK). The size and polydispersity index (PDI) of lipoplexes were measured in three biological replicates by dynamic light scattering (DLS) on a Malvern Zetasizer Nano instrument (Malvern Instruments, Malvern, UK) at a 173° scattering angle and 25 °C. The measurements were performed in Malvern’s Zetasizer v7.11 software (Malvern Instruments). A viscosity of 0.8872 centiposes (cP), a refractive index (RI) of 1.330 for the dispersant, and an RI of 1.020 and absorption of 1.335 for the material in suspension were chosen as settings in the software. An equilibration duration of 30 s was selected before the total measurement. ζ-Potential was measured at 25 °C in three biological replicates. Before the measurement, the equilibration duration was set to 120 s. Each measurement was paused for 30 s before the next one.

Atomic force microscopy (AFM) imaging. AFM images were captured in ambient air. Sample preparation for AFM was as follows: (1) dilution of samples to desired concentration, (2) deposition of 6 μL of a sample onto a freshly prepared mica slide (1 × 1 cm) for adsorption for 60 s, (3) rinsing with 100–1,000 μL of MilliQ water, and (4) drying the specimen with a gentle argon stream. Images were acquired on a Multimode 8 (Bruker) atomic force microscope in “ScanAsyst in Air” mode using ScanAsyst-Air probes (Bruker) or in tapping mode with a diamondlike carbon NSG-10 series AFM cantilever (NT-MDT, Zelenograd, Russia) having a tip curvature radius of 1–3 nm. Images were processed, prepared, and analyzed in the Gwyddion software.Cell lines. The HEK293T/17 cell line was purchased from ATCC (cat. # CRL-11268). Cells were cultured at 37 °C in the DMEM/F12 (1:1) medium supplemented with 10 % of fetal bovine serum (FBS), 1× sodium pyruvate, 1× GlutaMax, 1× antibiotic/antimycotic, and 1× non-essential amino acids (all solutions from Gibco, USA) in a humidified atmosphere with 5 % of CO2.

Cell transfection in vitro. The transfection was performed on HEK293T/17 cells. For the assay, cells were seeded in 24-well plates at 1.4 × 105 cells/well and cultured to 60–70 % confluence in the medium described above. To avoid the degradation of RNA in the lipoplexes, the FBS-containing culture medium was replaced with the 450 μL/well of FBS-free culture medium (the cells were washed with PBS in-between). For the formation of lipoplexes, both RNA (500 ng per well) and liposomes 2X3-DOPE (1:3) were diluted with PBS to a volume of 25 μL per sample with their subsequent mixing. The mixture was incubated for 20 min for lipoplex formation. The lipoplexes were added to the FBS-free cell medium, and the transfection lasted for 5 h. To stop the transfection, the FBSfree medium was replaced with the FBS-containing medium (with intermediate washing with PBS).

Flow cytometry. The transfection of GFP mRNA was carried out as described above in 24-well plates. At 24 h posttransfection, the cells were detached with TrypLE (Gibco, USA), centrifuged for 5 min at 500 × g, washed with PBS once, and resuspended in 1 mL of PBS containing 0.5 % of FBS. To assess the level of GFP expression, 10,000 events per sample were acquired on a BD FACSCanto II flow cytometer (BD Biosciences, USA). Transfection efficiency was measured by flow cytometry with the help of two parameters: transfection percentage and mean fluorescence intensity (MFI). The transfection percentage was calculated as the percentage of GFP-positive singlets. The MFI was computed as the mean for a gated cell population. The results were analyzed in the FlowJo software and are presented as the mean and standard deviation (SD) from three replicates.

The time course of luminescence detection in vitro. The transfection of Fluc2 mRNA was carried out as described above in 24-well plates. 24h post-transfection medium was removed and 200 μL of cold Luciferase Assay Buffer (25 mM Tris-HCl pH 7.8, 1 % Triton-X100, 5 mM EDTA, 15 mM MgCl2, 75 mM NaCl, 2 mM DTT, 2 mM ATP) was added. The plates were incubated at +4 °C for 20 min. After lysis, the suspension from each well was centrifuged in a separate 1.5 mL tubes at +4 °C, 12,000 g, 5 min, then 190 μL of each supernatant was transferred into a new 24-well plate. The luminescence level (represented in relative luminescence units, RLU) was measured with ClarioStar Plus (BMG Labtech, Germany) after injecting 10 μL of 3 mg/mL D-luciferin substrate solution (D-luciferin Potassium Salt, GoldBio, USA) per well. The data were analyzed in BMG Labtech CLARIOstar MARS Software.The time course of luminescence detection in vivo. For in vivo experiments, female 4–6-week-old BALB/c mice were obtained from the vivarium of the ICBFM SB RAS (Novosibirsk, Russia). The animal experiments were conducted in accordance with the recommendations for the proper use and care of laboratory mice (ECC Directive 2010/63/EU). All experimental protocols were approved by the Animal Research Ethics Committee at the Institute of Cytology and Genetics SB RAS (Novosibirsk, Russia) (protocol No. 173 of 7 May 2024).

The experiments with mice were conducted in three biological repeats. BALB/c mice were intramuscularly (i. m.) injected with lipoplexes of FLuc mRNA with liposomes in PBS (N/P = 6/1, 10 μg of mRNA, 150 μL per animal). Luciferase expression in vivo was assessed 4, 8, 24, 48, 72, and 96 h after administration of lipoplexes to mice. The animals were injected intraperitoneally (i. p.) with 150 μL (3.6 mg per mouse) of freshly prepared D-luciferin potassium salt (Gold Biotechnology, CA, USA) in PBS. After 15 min, the animals were anesthetized with isoflurane, and bioluminescence was visualized using IVIS Lumina X5 (Perkin Elmer, Waltham, MA, USA) with an exposure time of 3 min. The intensity of the luminescent signals was estimated by densitometry using Living Image software v.4.7.4 (Perkin Elmer, Waltham, MA, USA).Statistical analysis. All data plotted with error bars are expressed as the mean with standard deviation, unless otherwise indicated. GFP signal data were analyzed using one way ANOVA, FLuc signal – using a two-tailed unpaired t-test. Significance was evaluated at p < 0.05.

## Results and discussion

**Construction and synthesis of the reporter mRNAs (GFP and FLuc mRNAs) with different types of UTRs**

To correctly evaluate the effectiveness of the mRNA delivery in different conditions, the mRNA structure containing 5′-cap, UTRs and poly(A)-tail was suggested. The artificial pseudouridinilated and capped mRNAs were synthesized from linearized plasmids coding for a respective RNA with subsequent T7-mediated transcription and purification (Fedorovskiy et al., 2024).

The abundance of the nucleotide modifications and the combination of modifications in mRNAs with different UTRs were typical (100 % substitution of uridine by pseudouridine); therefore, they did not affect the expression level when comparing mRNAs with different UTRs. For all mRNAs, polyadenylation was carried out under identical conditions, which also could not alter the expression level. Thus, the mRNA synthesis was varied only in terms of the UTR combination.

The purity and integrity of the synthesized in vitro polyadenylated mRNAs for the subsequent assays were tested by electrophoresis in a 1.5 % agarose gel (Supplementary Fig. S5).

**The physicochemical characterization of the liposomes and their complexes with reporter mRNAs**

Upon mRNA characterization, we tested the characteristics of their complexes with the lipid carriers (lipoplexes). The 2X3- DOPE composition at a 1:3 ratio (Fedorovskiy et al., 2024) was used as the carrier in this work as one of the most efficient liposomes tested in previous studies (Markov et al., 2015; Gladkikh et al., 2021), particularly due to the positive impact of the high content of helper lipid DOPE on efficient lipoplex formation and delivery (Vysochinskaya et al., 2022). For the subsequent in vivo assays, the polyethyleneglycol (PEG)- decorated lipoconjugate was added to the liposome composition for more prolonged circulation in the blood stream and better clearance. The N/P ratios of 4/1 for the in vitro and 6/1 for the in vivo delivery were used. Physicochemical properties of the lipoplexes containing FLuc mRNA were examined, including hydrodynamic diameters and ζ-potentials of the liposomes and lipoplexes as described in (Fedorovskiy et al., 2024) (see the Table).

**Table 1.**
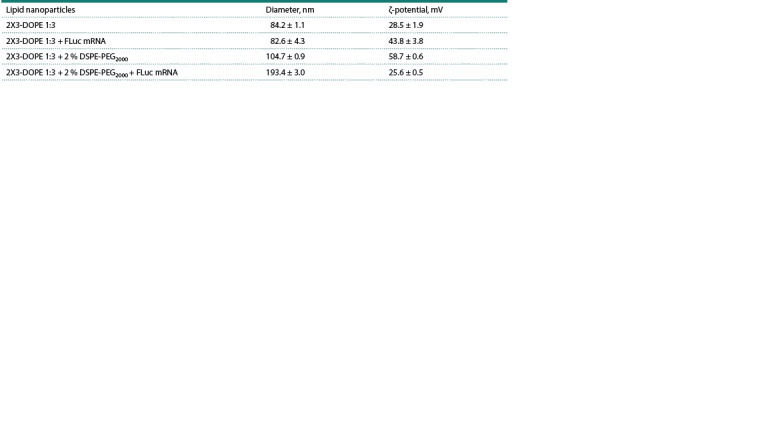
Diameters and ζ-potentials of 2X3-DOPE 1:3 and 2X3-DOPE 1:3 + 2 % DSPE-PEG2000 liposomes and their lipoplexes with FLuc mRNA (synthetic UTRs) Note. The data are presented as the mean ± SD of three replicates.

It was shown that the formed lipoplexes were characterized by a small diameter of <200 nm and a positive surface charge of +25…+45 mV, which facilitates their permeabilization through the cell membrane. Additionally, the diameters of the lipoplexes were evaluated by AFM (see Supplementary Fig. S6). The lipoplexes were shown to form homogenous nanoparticles sized 100–200 nm, which confirms the dynamic light scattering measurements. The results demonstrated that the characteristics of the formed lipoplexes were appropriate for in vitro and in vivo delivery (Vysochinskaya et al., 2022; Fedorovskiy et al., 2024).

**The in vitro comparison of reporter mRNA expression levels**

**Fig. 2.**
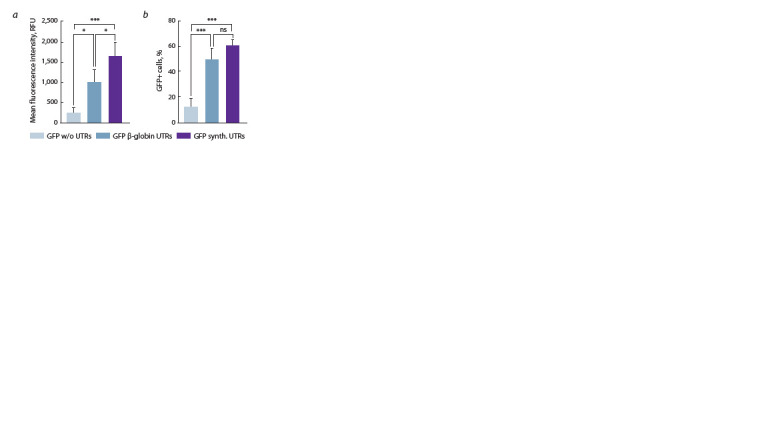
The comparative analysis of fluorescent signal intensity in GFP mRNA transfected HEK293T/17 cells. a, The mean fluorescence intensity (RFU) in transfected cells; b, the proportion of GFP-positive cells in transfected cells. The data are shown as the mean ± SD of three biological replicates. Data were statistically analyzed using ordinary one-way ANOVA. * p = 0.0191; 0.0474 for comparison of cells transfected with GFP with β-globin UTRs or GFP mRNA without UTRs; cells transfected with GFP with β-globin UTRs or GFP with synthetic UTRs respectively (mean GFP+ RFU); *** p = 0.0009; 0.0005; 0.0001 for comparison of cells transfected with GFP with synthetic UTRs or GFP mRNA without UTRs (mean GFP+ RFU); GFP with β-globin UTRs and GFP without UTRs; GFP with synthetic UTRs and GFP without UTRs respectively (GFP-positive cells, %), respectively; ns – for comparison of cells transfected with GFP with β-globin UTRs or GFP with synthetic UTRs (GFP-positive cells, %).

To identify the most effective mRNA structure upon lipoplex delivery, two mRNA models, namely, GFP and FLuc, were used to test the reporter protein expression in vitro on HEK293T/17 cells. Initially, we examined expression efficiency of GFP mRNAs constructed with either β-globin or synthetic UTRs. To confirm the necessity of 5′- and 3′-UTRs in mRNA structure, we estimated the expression level for mRNAs without UTRs in the same experiment (Fig. 2).

The crucial role of UTRs for the prominent expression of synthetic mRNA was confirmed by the increase by 3.9– 4.7 times in the number of GFP-positive cells and a 1.5–2.0- fold increase in the mean fluorescence intensity after the addition of UTRs to the mRNA structures. The fluorescence assay revealed a 1.5-fold increase in the fluorescence level of the cells transfected with mRNAs containing the novel UTR combination as compared to mRNAs carrying the commonly used β-globin UTRs. Moreover, this finding supports the idea of more effective expression due to the higher ribosome load at the 5′ end of mRNA (Orlandini von Niessen et al., 2019) and shows effective interaction of nucleotide motifs from these UTRs.

**Fig. 3.**
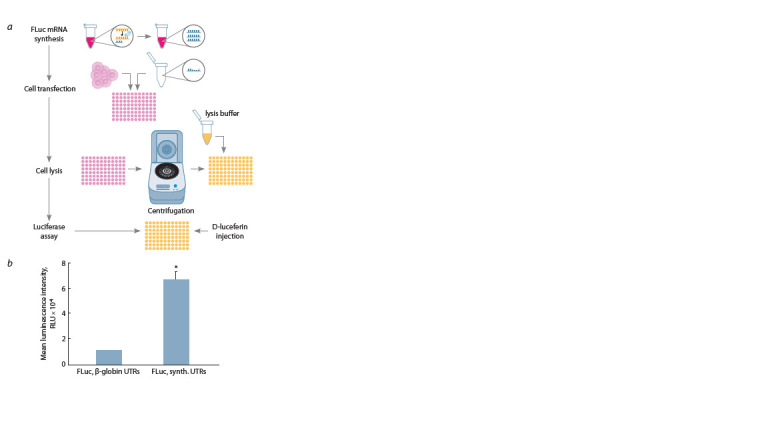
The luciferase assay and luminescence analysis of FLuc mRNA containing β-globin or synthetic UTRs. a, The schematic illustration of the luciferase assay in vitro. b, The average luminescent signals of the transfected cells. The data are presented as the mean ± SD of three biological replicates. Data were statistically analyzed using two-tailed Student’s t-test. * p = 0.00009 as compared with FLuc mRNA containing β-globin UTRs.

To further confirm the efficacy of the novel UTR combination in the translation of reporter mRNAs within cells, an alternative mRNA encoding FLuc was used. The results demonstrated that mRNA flanked by the synthetic UTRs exhibited a luminescent signal intensity that was 6–7 times greater than that observed in mRNA containing β-globin UTRs (Fig. 3b). The more sufficient growth of the specific signal in the luminescent assay could be explained by the more significant sensitivity of the luminescence detection (Troy et al., 2004). Indeed, the luminescence detection tends to be 100-fold more sensitive than the commonly investigated fluorescent detection; thus, the result allows a more precise evaluation of the comparative effectiveness of the investigated combination of UTRs. On the other hand, the higher signal for the luminescence assay upon the substitution of the β-globin UTRs with synthetic UTRs could be caused by the higher expression level of the FLuc gene rather than GFP. Mrksich et al. showed that the longer hydrophobic tail of the cationic lipid facilitates the higher translation level of the more prolonged mRNA. As 2X3 used in our assays is quite compact and less branched even compared with C12-200 (Mrksich et al., 2024), it promotes the higher expression of FLuc mRNA with more prolonged UTRs.

**The in vivo comparison of reporter mRNA expression levels**

**Fig. 4.**
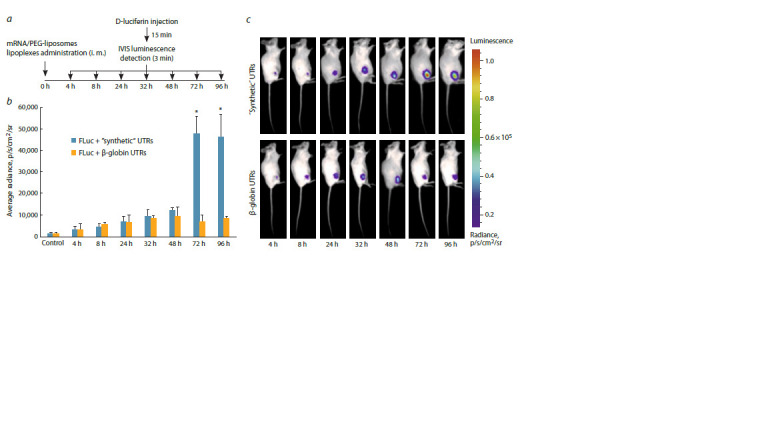
In vivo luminescence analysis of BALB/c mice injected with FLuc mRNA. a, The scheme of the experiment with key time intervals; b, the mean luminescence intensities of regions of interest (ROIs) in mice. The blue and orange bars represent the mice immunized with the FLuc mRNA containing synthetic or β-globin UTRs, respectively. The results are presented as the mean ± SD of three replicates. c, Representative IVIS images of Fluc luminescence in mice after the injection of lipoplexes. Data were statistically analyzed using two-tailed Student’s t-test. * p = 0.0001 compared with FLuc mRNA containing β-globin UTRs.

To evaluate the influence of different UTRs on the efficiency of mRNA translation in vivo, lipoplexes of PEGylated liposomes with FLuc mRNAs containing either β-globin or synthetic UTRs were intramuscularly injected into BALB/c mice (Fig. 4).

The in vivo results demonstrated that mRNA containing a novel combination of 5′-UTR-4 and 3′-UTR AES-mtRNR1 exhibited dramatically elevated luminescence signal at late time points (≥72 h post injection) that was six times higher compared to β-globin-UTR-containing mRNA. In vivo findings revealed a dramatic increase in the luminescent signal observed at 72 h post injection, whereas for the β-globin UTRs, the specific signal tended to decay at 48 h post injection. It is worth noting that in our study, the signal peak shifted from several hours post injection (as shown e. g. (Panova et al., 2023)) to 48 h post injection and later. This shift could be caused not only by the carrier molecules used in this work and their properties but also by the specific translation pattern resulting from the novel UTR combination. According to (Ruis de los Mozos et al., 2013), 5′ and 3′ components of mRNA tend to interact with each other, providing stabilization of the mRNA. Moreover, the extension of 3′-UTR length could have a positive effect on the half-life of the mRNAs through interactions with RNA-binding proteins. The specific luciferase signal was detected even 174 h post injection of artificial mRNA with the novel UTR combination (Supplementary Fig. S7), which may indicate a longer half-life of the mRNA. These results really merge with the in vitro assays, indicating the advantages of the novel UTR combination. Long-term presence of mRNA in mammalian tissues and long-term expression of the target gene have been previously described, but for other delivery systems, which explains the difference between our data and other studies (Hassett et al., 2024). The expression enhancement accomplished in our research may facilitate the development of antiviral or anticancer mRNA vaccines possessing higher immunogenicity than the existing analogues.

## Conclusion

Overall, the results of this study indicate that the novel combination of synthetic 5′-UTR-4 and 3′-UTR AES-mtRNR1 UTRs introduced into reporter mRNAs demonstrated enhanced mRNA translation in comparison with mRNA containing β-globin UTRs in both in vitro and in vivo experiments. The optimization of the mRNA structure should improve the development of effective antiviral and anticancer mRNA modalities, which can compete with other types of vaccines and therapeutics.

## Conflict of interest

The authors declare no conflict of interest.
